# Gastroduodenal Morphology and Related Symptoms in Chronic Alcoholics

**DOI:** 10.1155/DTE.4.29

**Published:** 1997

**Authors:** T. Hauge, J. Persson, Å. Nilsson

**Affiliations:** 1 Department of Internal Medicine Central Hospital Karlstad Sweden; 2 Department of Internal Medicine Lund University Hospital Sweden; 3 Department of Internal Medicine Central Hospital of Østfold Fredrikstad N-1603 Norway

## Abstract

Twenty-four chronic alcoholics admitted to hospital for detoxification after a drinking spree were examined by upper gastrointestinal endoscopy. Biopsy specimens were taken from corpus/fundus, antrum and duodenum for tissue histology (eosin stain). From the duodenum villus index and ultrastructure (scanning electron microscopy, SEM) were also performed. As a control group 12 subjectively healthy non-alcoholics referred to upper gastrointestinal endoscopy mainly for dyspepsia were chosen.

Gastrointestinal symptoms were common in alcoholics (88%). Endoscopic and histological gastroduodenitis were not more common in the alcohol group. There was no correlation between gastrointestinal symptoms and endoscopic or histological gastroduodenitis in both groups. In the duodenum, 50% of the alcoholics and 82% in the control group had alterations by scanning electron microscopy. Ten of the 11 alcoholics with an abnormal ultrastructure had diarrhoea. In the control group dyspepsia (ulcus suspect) was correlated to a pathological SEM.


*Diagnostic and Therapeutic Endoscopy*, Vol. 4, pp. 29–33
Reprints available directly from the publisher
Photocopying permitted by license only

© 1997 OPA (Overseas Publishers Association)
Amsterdam B.V. Published in The Netherlands
by Harwood Academic Publishers
Printed in Singapore

### Gastroduodenal Morphology and Related Symptoms in Chronic Alcoholics

T. HAUGE[a,*], J. PERSSON[a] and Å. NILSSON[b]

[a]*Department of Internal Medicine, Central Hospital Karlstad, Sweden;* [b]*Department of Internal Medicine, Lund University Hospital, Sweden*

*(Received 17 January 1997; In final form 24 February 1997)*

**Twenty-four chronic alcoholics admitted to hospital for detoxification after a drinking spree were examined by upper gastrointestinal endoscopy. Biopsy specimens were taken from corpus/fundus, antrum and duodenum for tissue histology (eosin stain). From the duodenum villus index and ultrastructure (scanning electron microscopy, SEM) were also performed. As a control group 12 subjectively healthy non-alcoholics referred to upper gastrointestinal endoscopy mainly for dyspepsia were chosen.**

**Gastrointestinal symptoms were common in alcoholics (88%). Endoscopic and histological gastroduodenitis were not more common in the alcohol group. There was no correlation between gastrointestinal symptoms and endoscopic or histological gastroduodenitis in both groups. In the duodenum, 50% of the alcoholics and 82% in the control group had alterations by scanning electron microscopy. Ten of the 11 alcoholics with an abnormal ultrastructure had diarrhoea. In the control group dyspepsia (ulcus suspect) was correlated to a pathological SEM.**

*Keywords:* Alcoholics, Gastrointestinal symptoms, Upper gastrointestinal endoscopy, Tissue pathology, Villus index, Scanning electron microscopy, SEM

### INTRODUCTION

Gastrointestinal symptoms like abdominal pain/ dyspepsia, diarrhoea and nausea are common among individuals with chronic alcohol ingestion. Morphological alterations and functional disturbances in the upper gastrointestinal tract can also be related to alcohol abuse [1–3].

The acute alcohol induced gastritis, or better subepithelial hemorrhages, which disappears within a few days is well known [4,5]. Similar acute findings are found in the duodenum [1,6,7].

The chronic effects on the gastroduodenal morphology have not been extensively studied. A proven correlation between alcohol and chronic gastritis has not been found [4,8]. In the small intestine the effects of chronic alcohol abuse on the morphology are conflicting. Usually duodenal and jejunal biopsies have showed normal histology, but a reduction in villus height in relation to

* Corresponding author. Present address: Department of Internal Medicine, Central Hospital of Østfold, N-1603 Fredrikstad, Norway. Tel.: 47 69 39 3000. Fax: 47 69 39 3269.

crypt depth has been observed [2,9–12]. It is therefore possible that chronic alcohol consumption in man induces slight histological changes. Studies on the ultrastructure in the small intestine have showed pronounced ultrastructural alterations [3,10,13]. Only a few studies have been performed on the duodenal mucosa by gastrointestinal endoscopy.

The aim of the present study was to investigate the effects of chronic alcohol ingestion on the gastroduodenal mucosa by endoscopy, light microscopy and ultrastructure (scanning electron microscopy, SEM). The morphological findings were also compared to gastrointestinal symptoms.

### MATERIAL AND METHODS

Twenty-four chronic alcoholics (5 women and 19 men) admitted to hospital for detoxification after a drinking spree were examined with upper gastrointestinal endoscopy as soon as possible. Mean average age was 46 years (range 33–59). The endoscopical examination was performed 4 days (range 2–7) after last alcohol intake. Mean alcohol consumption was 338 g pure alcohol/day (range 102–680) in the weeks before admission. The alcoholics were questioned about past medical history, alcohol consumption, present symptoms with particular regard to diarrhoea, nausea and abdominal pain and physically examined directly after admission. Diarrhoea was defined as abnormal, frequent, loose stools. Patients with manifest alcohol related somatic diseases, peptic ulcers, malnutrition or use of drugs known to affect intestinal integrity, were excluded. The smoking incidence in the alcohol group was 100%. None were treated with gastric acid inhibitors at the time for examination.

The control group consisted of individuals referred for upper endoscopy mainly for dyspepsia. They were 12 individuals (2 women, 10 men) with mean average age 42 years (range 24–72). These were subjectively healthy, had no or a very little alcohol consumption and had no gross pathological changes by the upper endoscopy. Seven controls had been treated with gastric acid inhibitors.

The biopsy specimens were performed with an Olympus FB-24K forceps by one and the same endoscopist (TH). Biopsies were taken from corpus/fundus, antrum and the distal part of the descending duodenum for light microscopy. From the duodenum two biopsies were also taken for scanning electron microscopy (SEM). For the light microscopy a haematoxylin/eosin stain was performed. An experienced pathologist examined all biopsy specimens having no information about the patients. The villus index was defined as the ratio between height of the villi plus the depth of the crypts (from the bottom of the crypts up to the tip of the villi) over the depth of the crypts [10]. Thus, a flat mucosa will give a mucosal index of 1.0, and a normal mucosa could give an index up to 6.

For the electron microscopy (SEM) the biopsies were immediately fixed in 4% glutaraldehyde solution in 0.1 M phosphate buffer (pH 7.2), rinsed carefully with 0.9% NaCl, dehydrated in rising concentrations of ethanol, dried and coated with gold in a combined system with a sputter (Edwards S 150 A sputter coating unit) and a modified vacuum unit (Edwards vacuum coating unit, 140 d E12–E14). Finally it was examined in a Cambridge Stereoscan 360 scanning electron microscope. Each specimen was studied at 3 different magnifications assessing the structural architecture of the small intestine (×100, ×1000, ×10 000).

*Statistical method*: Wilcoxon's rank sum test. The study was approved by the Local Ethical Committee and informed consent was obtained from all patients.

### RESULTS

*Symptoms*: Twenty-one of 24 (88%) alcoholics had gastrointestinal symptoms like abdominal pain, diarrhoea and nausea. Abdominal pain/dyspepsia was the most predominant symptom, fourteen patients (58%) had this symptom, twelve patients (50%) had diarrhoea by the examination. Among the controls 6 (50%) had abdominal pain/dyspepsia, none had diarrhoea.

*Endoscopy*: In the alcohol group only three had gastritis (12%) with subepithelial hemorrhages at he upper endoscopy. Twelve (50%) had a mild gastritis and nine (38%) alcoholics had a normal upper endoscopy. In the control group seven (58%) had a mild gastritis and five (42%) upper endoscopies were normal. The endoscopy did not demonstrate a difference between the two groups. A duodenitis was seen in two (8%) of the alcoholics and in none control patient by endoscopy.

*Morphology*: The histological examination in the alcohol group showed antral gastritis in six alcoholics (25%). Two (8%) had a corpus/fundus gastritis and 17 (71%) were normal. Helicobacter pylori was found in 7 (29%) according to culture. In the control group five (42%) had an antral gastritis and two (17%) had a corpus/fundus gastritis. Four of twelve (33%) were Helicobacter pylori positive. Six (50%) were histologically normal.

The histological examination demonstrated a duodenitis in eight (33%) alcoholics and in three (25%) of the controls.

There was no correlation between symptoms and findings by the upper endoscopy or the histological examination whether in alcoholics nor in the control group. For symptoms and histology, see also Table I.

In the duodenum the mucosal index was examined. The mean value in the alcohol group was 3.15±0.15 SEM compared to 3.32±0.20 SEM

TABLE I The relation between symptoms, morphology and alcohol consumption in alcoholics

| Pat. | Symptom | (Light microscopy) Stomach | Duodenum (villus index) | Electronmicro (SEM) | Alcohol (g/day) |
|---|---|---|---|---|---|
| 1 | no | normal | 4.00 | — | 360 |
| 2 | no | normal | 3.75 | normal | 240 |
| 3 | no | normal | 3.81 | normal | 240 |
| 4 | D | normal | 2.75 | normal | 160 |
| 5 | P | m.a.g. | 3.45 | normal | 385 |
| 6 | D, P, N | normal | 2.57 | path. | 324 |
| 7 | D, N | normal | 2.13 | path. | 108 |
| 8 | P, N | normal | 3.40 | normal | 328 |
| 9 | P | gastritis | 3.17 | normal | 680 |
| 10 | D, P | normal | 2.40 | path. | 450 |
| 11 | D, P | normal | 1.44 | path. | 640 |
| 12 | N | normal | 4.10 | normal | 480 |
| 13 | D | normal | 3.38 | path. | 328 |
| 14 | D | normal | 3.45 | normal/bact. | 204 |
| 15 | P, N | a.g. | 4.09 | — | 570 |
| 16 | D | normal | 3.43 | path. | 353 |
| 17 | D, P, N | m.a.g. | 3.86 | path. | 480 |
| 18 | P | carcinoma | 2.29 | (normal) | 108 |
| 19 | P | normal | 3.55 | normal/bact. | 102 |
| 20 | D, P | normal | 3.07 | path./bact. | 125 |
| 21 | P | normal | 3.75 | path. | 360 |
| 22 | D, P | normal | 3.21 | path. | 370 |
| 23 | P | gastritis | 2.34 | normal | 360 |
| 24 | D, P | a.g. | 2.15 | path. | 350 |
| | | | 3.15 (±0.15 SEM) | | 338 (102–680) |

D = diarrhoea, P = abdominal pain, N = nausea, m.a.g. = mild antrum gastritis, a.g. = antrum gastritis.

in the control group, what means a slightly, not significant, reduction ($p$ = NS).

*Ultrastructure (SEM)*: Eleven of 22 alcoholics (50%) had pathological scanning electron microscopy: decreased glycocalyx layer and irregularities in the microvilli. Two biopsies could not be examined. There was a good correlation between these findings and diarrhoea (10 of 11 had diarrhoea) in the alcohol group and four of these 11 alcoholics (36%) had a histological duodenitis. The alcoholics with pathological scanning electron microscopy did not have a higher alcohol consumption than the other. By dividing the alcohol group into pathological and normal ultrastructure, there was no correlation between villusindex and ultrastructural findings.

*Controls*: Nine of eleven (82%) had a pathological SEM, one of the controls could not be examined. There was a correlation between ultrastructure and dyspepsia (ulcus suspect). Seven of 11 (64%) controls were referred to upper gastrointestinal endoscopy for dyspepsia. Of the two controls with previous dyspepsia one had received pharmacological treatment for ulcus ventriculi, one was operated for a hiatus hernia and came for a one year follow up endoscopy. The two controls wth normal SEM in the duodenum could not remember any episodes of dyspepsia (ulcus suspect).

### DISCUSSION

The present study shows that gastrointestinal symptoms like abdominal pain/dyspepsia and diarrhoea are common in alcoholics. Gastroduodenitis was not more frequent among excessive alcoholics compared to a control group. Half of the alcohol group and 82% of the control group had electron microscopical alterations, and there was a correlation between these findings and symptoms.

A slightly, not statistical significant, reduced mucosal index in the duodenum compared to controls was also observed. The mean value of the mucosal index ($3.15 \pm 0.15$ SEM) was almost the same as found in biopsies taken by a Watson capsula ($3.18 \pm 0.21$ SEM) from the upper jejunum in another study [10]. This finding support the few previous studies of alcohol effects on the duodenal mucosa [2].

Fifty percent of the alcoholics had pathological ultrastructure by scanning electron microscopy. In a previous study, 70% had SEM alterations after chronic alcohol intake. It is possible that alcohol effects on the duodenum differs from the jejunum. The ultrastructural changes correlated to diarrhoea (10 of 11 had diarrhoea), accepted as one of the main consequences of acute and chronic high alcohol intake [14]. The frequency of SEM alterations was surprisingly higher in the control group. Comparable studies from a normal population does not exist, but control biopsies from healthy individuals have showed normal ultrastructure [8].

Our control group was a small group mainly with dyspepsia (ulcus suspect). Therefore the controls are not identical with a perfectly healthy control group. On the other hand, it would be difficult to recruit individuals free from symptoms for a upper gastrointestinal endoscopy.

The poor correlation between endoscopic and histological findings is well known from other studies [2,15].

In conclusion, the chronic alcohol effects on the gastroduodenal mucosa are in accordance with previous studies. Alcohol causes slight changes in mucosal index in the duodenum, and more pronounced ultrastructural alterations. Diarrhoea had a correlation to ultrastructural alterations in the alcohol group. Dyspepsia had the same correlation in the control group. These observation requires further investigation.

### *Acknowledgements*

Thanks to R. Stenling M.D. Ph.D. Department of Pathology, Umeå University Hospital, Sweden.

Financial support was given by the Swedish Medical Society and by the Centre of Public Health Research, Karlstad, Sweden.

### *References*

[1] Persson, J. Alcohol and the Small Intestine. *Scand. J. Gastroenterol.* 1991; **26**: 3–15.

[2] Lev, R. *et al.* Pathological and Histomorphometric Study of the Effects of Alcohol on the Human Duodenum. *Digestion* 1980; **20**: 207–213.

[3] Rossi, M.A. and Zucoloto, S. Effect of Chronic Ethanol Ingestion on the Small Intestinal Ultrastructure in Rats. *Beitr. Path.* 1977; Vol. 161, 50–61.

[4] Wolff, G. Alkoholwirkung am Magen. *Gastroenterol. J.* 1989; **49**: 45–49.

[5] Laine, L. and Weinstein, W.M. Histology of Alcoholic Hemorrhagic "Gastritis": A Prospective Evaluation. *Gastroenterology* 1988; **94**: 1254–62.

[6] Gottfried, E.B., Korsten, M.A. and Lieber, C.S. Alcohol-induced Gastric and Duodenal Lesions in Man. *American Journal Gastroenterology* 1978; **70**: 587–592.

[7] Beck, I.T. and Dinda, P.K. Acute Exposure of Small Intestine to Ethanol. Effects on Morphology and Function. *Digestive Diseases and Sciences* (September 1981); Vol. 26, No. 9.

[8] Bode, J.Ch. Alcohol and the Gastrointestinal Tract. *Advances in Internal Medicine and Pediatrics.* In Frick *et al.* Springer Verlag 1980.

[9] Pirola, R.C., Bolin, T.D. and Davis, A.E. Does Alcohol Cause Duodenitis? *New Series*, 1969; Vol. 14, No. 4.

[10] Persson, J. *et al.* Morphologic Changes in the Small Intestine after Chronic Alcohol Consumption. *Scand. J. Gastroenterol.* 1990; **25**: 173–184.

[11] Sjölund, K., Persson, J. and Bergman, L. Can villous atrophy be induced by chronic alcohol consumption? *Journal of Internal Medicine* 1989; **226**: 133–135.

[12] Bode, J.Ch. *et al.* Quantitative Histomorphometric Study of the Jejunal Mucosa in Chronic Alcoholics. *Digestion* 1982; **23**: 265–270.

[13] Rubin, E. *et al.* Ultrastructural Changes in the Small Intestine Induced by Ethanol. *Gastroenterology* 1972; Vol. 63, No. 5.

[14] Burbige, E.J., Lewis, Jr. D.R. and Halsted, C.H. Alcohol and the Gastrointestinal Tract. *Medical Clinics of North America* (January 1984); Vol. 68, No. 1.

[15] Jönsson, K.-Å., Gotthard, R., Bodemar, G. and Brodin, U. The Clinical Relevance of Endoscopic and Histologic Inflammation of Gastroduodenal Mucosa in Dyspepsia of Unknown Origin. *Scand. J. Gastroenterol.* 1989; **24**: 385–395.

